# Immunomodulatory capacity of the serotonin receptor 5-HT2B in a subset of human dendritic cells

**DOI:** 10.1038/s41598-018-20173-y

**Published:** 2018-01-29

**Authors:** Attila Szabo, Peter Gogolak, Gabor Koncz, Zsofia Foldvari, Kitti Pazmandi, Noemi Miltner, Szilard Poliska, Attila Bacsi, Srdjan Djurovic, Eva Rajnavolgyi

**Affiliations:** 1 NORMENT, KG Jebsen Centre for Psychosis Research, Institute of Clinical Medicine, University of Oslo, Oslo, Norway; 2 Division of Mental Health and Addiction, Oslo University Hospital, Oslo, Norway; 3 Department of Immunology, Faculty of Medicine, University of Debrecen, Debrecen, Hungary; 4 Department of Biochemistry and Molecular Biology, Faculty of Medicine, University of Debrecen, Debrecen, Hungary; 5 NORMENT, KG Jebsen Centre for Psychosis Research, Department of Clinical Science, University of Bergen, Bergen, Norway; 6 Department of Medical Genetics, Oslo University Hospital, Oslo, Norway

## Abstract

Serotonin is a monoamine neurotransmitter that signals through a wide array of receptors (5-HT_1–7_) many of which are also involved in immune processes. Dendritic cells (DCs) are crucial players in immune defense by bridging innate and adaptive immune responses via their vast repertoire of pattern recognition receptors and antigen-presenting capability. Although serotonin is known to influence immunity at many levels, cell type-specific expression and function of its receptors remains poorly understood. Here we aimed to study 5-HT_1–7_ expression and function in CD1a^−^ and CD1a^+^ human monocyte-derived DCs (moDCs). We found that the 5-HT_2B_ receptor-subtype is solely expressed by the inflammatory CD1a^+^ moDC subset. Specific 5-HT_2B_ activation potently inhibited TLR2, TLR3, and TLR7/8-induced proinflammatory cytokine and chemokine (TNF-α, IL-6, IL-8, IP-10, IL-12) but not type I interferon-β responses. 5-HT_2B_ agonism also interfered with the polarization of CD1a^+^ moDC-primed CD4^+^ T cells towards inflammatory Th1 and Th17 effector lymphocytes. Here we report the subset-specific expression and immunomodulatory function of 5-HT_2B_ in human moDCs. Our results expand the biological role of 5-HT_2B_ which may act not only as a neurotransmitter receptor, but also as an important modulator of both innate and adaptive immune responses.

## Introduction

Dendritic cells (DCs) represent a diverse population of myeloid cells in higher vertebrates which play a crucial role in bridging innate and adaptive immunity in multiple tissue types. They fine-tune and control immune responses ensuring the maintenance of self tolerance as well as modulating lymphocyte functions by priming naive T cells and thereby contributing to the establishment of effector and memory subsets. Tissue resident DCs, by means of their diverse range of pattern recognition receptors (PRRs), continuously monitor their environment assessing the molecular composition of the given tissue^1^. PRRs can detect both external, pathogen-derived stimuli, such as the evolutionally conserved pathogen-associated molecular patterns (PAMPs), or self-derived endogenous danger signals (DAMPs) that are released during stress events. The ligation of PRRs usually leads to DC activation triggering the release of cytokines and chemokines, a phenomenon which is highly dependent on the nature of the stimulus, the surrounding tissue microenvironment and the participating PRR or cross-talk of PRRs, such as Toll-like receptors (TLRs) or RIG-I-like receptors (RLRs)^2^. This event leads to acute inflammatory and/or interferon responses through the mobilization of downstream signaling by nuclear factor kappa-B (NF-κB) and interferon regulatory factors (IRFs), respectively. This is followed by the recruitment of other innate immune cells to the site of activation and, via antigen-presentation, the orchestration and polarization of T cell responses^3^.

The monoamine neurotransmitter serotonin (5-hydroxytryptamine, 5-HT) is derived from L-tryptophan and is primarily found in the central nervous system (CNS), blood platelets, and gastrointestinal (GI) tract of animals. Most of the human body’s total serotonin is located within the GI tract produced and released by enterochromaffin cells; a significant amount of this 5-HT is absorbed and stored by platelets and, to a lesser extent, by other elements of the blood including lymphocytes, monocytes, and monocyte-derived cells^4^. Approximately 10% of the total 5-HT is synthesized in the CNS by serotonergic neurons where it exerts various functions, such as the regulation of mood, cognition, sleep, and appetite. The signaling of serotonin involves a wide array of serotonin receptors (5-HT_1–7_), which are dominantly G protein-coupled (GPCR) superfamily members with the exception of 5-HT_3_, a ligand-gated ion channel. GPCR 5-HT receptors signal by means of intracellular second messengers including MEK-ERK1/2 and the modulation of intracellular Ca^2+^ levels as downstream signals^5^.

Apart from its role in regulating gastrointestinal motility (GI tract), vasoconstriction, blood clotting, hemostasis (cardiovascular system), mood and cognition (CNS), serotonin is also involved in the regulation of inflammation and immune functions via controlling the release of cytokines and chemokines in a cell type-dependent manner^6,7^. Upon stimulation by LPS and IFNγ, both lymphocytes and monocytic cells release serotonin^8^. 5-HT, at normal tissue concentrations, is able to inhibit LPS-induced inflammatory responses (IL-1β, IL-6, TNF-α, CXCL8/IL-8, and IL-12 release) by human monocytes and PBMC^9,10^. Serotonin has also been shown to influence the differentiation capacity of human monocytes to dendritic cells, and modulate DC functions by increasing the release of the anti-inflammatory cytokine IL-10^11^. Moreover, 5-HT plays and important co-stimulatory role in the immunological synapse between DCs and T cells where it increases T cell activation mainly through the 5-HT_7_ subtype^12^ pointing to its importance in shaping the course of both innate and adaptive immune responses. Human DCs express the mRNA of several 5-HT receptor types with differential expression profile in resting (immature) and activated (mature) DCs, furthermore, 5-HT_4_ and 5HT_7_ receptor activation has been associated with altered cytokine release in mature dendritic cells^13^. In addition, previous reports linked the 5-HT_2_ and 5-HT_7_ receptor subtypes to anti-inflammatory functions and phenotype in human macrophages^7,14^. Altogether, these results suggest that 5-HT and its receptors modulate myeloid cell functions in a versatile way in human physiology.

Despite these earlier studies, the role of serotonin in DC biology remains poorly understood. Therefore, we aimed to examine the expression of 5-HT receptors and test the functional consequences of their activation in human CD1a^+^ monocyte-derived dendritic cells (moDCs) which has previously been described by our group as a functionally and phenotypically distinct, inflammatory DC subset^15–18^. Our major goal was to test the hypothesis that specific 5-HT receptor ligation can interfere with inflammatory reponses in this DC subset, which may as well expand our knowledge in the physiology and neuroimmunology of serotonin.

## Results

### Distinct expression pattern of 5-HT_2B_ in human dendritic cell subsets

**Figure 1:**
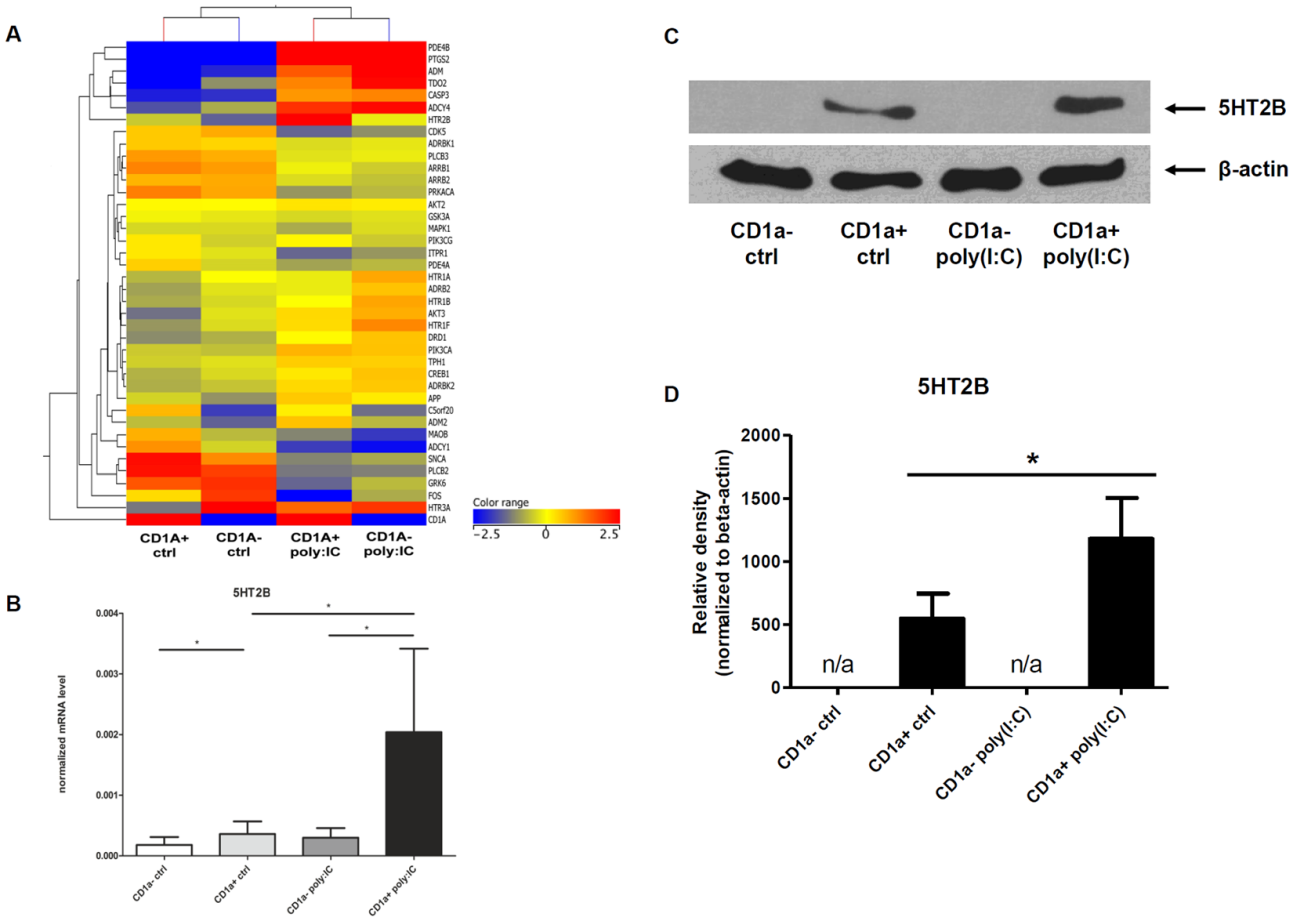
Distinct expression of 5-HT_2B_ in human monocyte-derived dendritic cell subsets MoDCs were differentiated in the presence of GM-CSF and IL-4 as described in Methods. (**A**–**B**) Heat map of relative mRNA expressions of serotonin receptors, transporters, metabolic enzymes and other factors involved in 5-HT signaling (panel ‘A’). Gene expressions of five independent donors were measured by TLDA array. Figure 1B represents normalized expression levels of 5-HT_2B_, values were extracted from the heat map on Fig. 1A. Mean ± SEM values of 5 donors are represented. (**C**) Western blot analysis of 5-HT_2B_ protein expression in CD1a^−^ and CD1a^+^ DCs. Activated cells were treated with 20 µg/ml polyI:C for 24 h before sampling. Results of a representative experiment out of 6 is shown. (**D**) Densitometry data show the Mean ± SEM values of 5-HT_2B_ protein expressions in six independent donors. Asterisk represents *p* values < 0.05.

The expression of 5-HT receptors in monocytes and monocyte-derived cells has been reported previously^7,13^. However, little is known about their expression and function in the functionally distinct subtypes of monocyte-derived DCs. Therefore we first examined the mRNA expression of serotonin receptors, 5-HT transporters, pathway elements involved in downstream signaling, as well as other factors that are involved in the cellular physiology of serotonin. We sought to investigate the expression of these 5-HT-related elements in the phenotypically and functionally different CD1a^+^ and CD1a^−^ DC subsets^15–18^ at baseline and in activated state using the dsRNA-mimic polyI:C, a synthetic ligand of the innate immune receptor TLR3^2,19^. We found statistically significant differences in the expression of five target genes in our TLDA array panel between the two DC subsets (HTR1A, HTR1F, HTR3A, 5HT2B, C5orf20; Figs 1 and S1). The degree of difference in mRNA expression was most marked in case of the 5-HT_2B_ receptor which showed significantly lower expression in CD1a^−^ compared to CD1a^+^ DCs (CD1a^−^ ctrl vs. CD1a^+^ ctrl, p < 0.05, n = 5). It was inducible upon 20 µg/ml polyI:C treatment in CD1a^+^ DCs (CD1a^+^ polyI:C vs. CD1a^+^ ctrl, p < 0.05, n = 5) but not in CD1a^−^ cells (CD1a^−^ poly: IC vs. CD1a^−^ ctrl, p > 0.05, n = 5) (Fig. 1). In line with these findings, the protein level of 5-HT_2B_ increased significantly in CD1a^+^ cells after activation by polyI:C, however we found no detectable levels of the receptor in CD1a^−^ cells either at baseline or post-activation as measured by western blot (Fig. 1C,D). This suggests a subset-specific expression of 5-HT_2B_ in human monocyte-derived dendritic cells.

### 5-HT_2B_ activation in human dendritic cells results in alterations of phenotypic and functional properties

**Figure 2:**
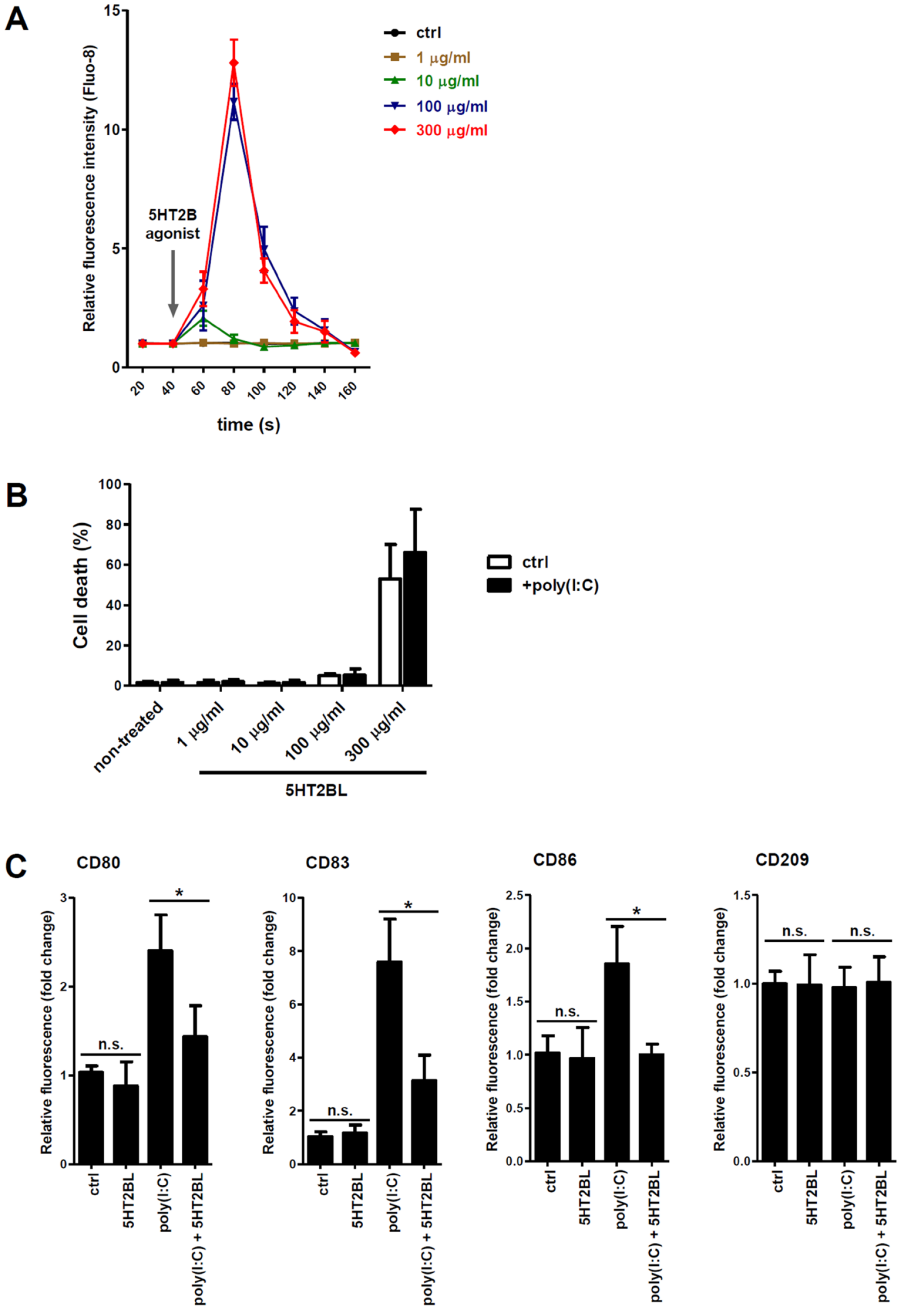
Specific activation of 5-HT_2B_ results in phenotypical and functional changes in CD1a^+^ DCs. CD1a^+^ moDCs were treated with 1–300 µg/ml of the selective 5-HT_2B_ receptor ligand BW723C86 hydrochloride (5HT2BL, 5-HT_2B_ agonist), and/or with 20 µg/ml polyI:C. (**A**) Changes in intracellular (IC) calcium levels were monitored by the fluorescence tracer Fluo-8 as in Methods. Time kinetics of IC Ca^2+^ alterations in non-activated control (ctrl) or in 1–300 µg/ml BW723C86 treated cells are shown. Data of triplicate measurements of four independent donors are represented as Mean ± SEM. (**B**) Rate of cell death following 5-HT_2B_ activation by increasing concentrations of BW723C86. Induction of apoptosis was evaluated by Annexin V-FITC staining after 24 h. Results of four independent donors are shown as Mean ± SEM; black bars mean 20 µg/ml polyI:C co-treated cultures whereas empty bars represent non-co-treated controls. (**C**) Following 12 h stimulation with 20 µg/ml polyI:C and/or 100 µg/ml BW723C86 (5HT2BL) the expression levels of CD80, CD83, and CD86 cell surface proteins were analyzed by flow cytometry. Relative fluorescence intensity values were calculated using the respective isotype-matched control antibodies. The bars represent fold changes compared to the untreated control (ctrl) and data are expressed as the Mean ± SEM of four (CD80, 83, 86) or three (CD209) independent experiments. *p < 0.05; *n*.*s*. means ‘non-significant’.

As the protein-level expression of 5-HT_2B_ was observable only in CD1a^+^ DCs (Fig. 1), we focused on this subset in our further studies. We first addressed the question whether 5-HT_2B_ is functional in CD1a^+^ cells, and if so, does its agonism cause any change in cellular phenotype and functions? 5-HT_2_ receptors utilize intarcellular Ca^2+^ mobilization as a major part of their downstream signaling. Thus, rapid elevations in cytoplasmic calcium levels subsequent to receptor ligation is regarded as a reliable indicator of 5-HT_2_ activation in many cell types^5,20^. In our studies we used BW723C86, a highly selective agonist of the 5-HT_2B_. Treatment of CD1a^+^ DCs by 1–300 µg/ml working concentrations of the agonist resulted in rapid intracellular Ca^2+^ signals as were monitored by the fluorescent reporter dye Fluo-8 (Fig. 2A). Calcium signals were peaking at both 100 and 300 µg/ml with no significant difference between the two concentrations (Fig. 2A). We also tested the possible cytotoxicity of BW723C86 and found that the 100–300 µg/ml cc range caused apoptotic cell death in the cell cultures after 24 h, and this effect was further potentiated by the inflammatory activator polyI:C (Fig. 2B). However, in case of the lower 100 µg/ml concentration, the ratio of dead cells never exceeded 6% (4 ± 2%, n = 4) even when co-administered with polyI:C (Fig. 2B), thus we decided to use the 100 µg/ml agonist concentration in our further experiments. We next checked whether the specific activation of 5-HT_2B_ leads to any change in the expression of cell surface markers critically involved in DC functions. Interestingly, expression of the activation markers CD80, CD83, and CD86, co-stimulatory molecules that play a pivotal role in DC-T cell communication, did not change following a 12 hours treatment with 100 µg/ml of BW723C86 (Fig. 2C). Neither did change the expression of the DC-specific C-type lectin receptor CD209/DC-SIGN (Fig. 2C). Mimicking viral infection we treated DCs with high-dose polyI:C to elicit strong inflammatory responses. In agreement with previous findings, polyI:C treatment resulted in upregulation of CD80, CD83, and CD86^21,22^. Remarkably, however, BW723C86 strongly inhibited the stimulatory effect of polyI:C on activation marker expression in co-treated cultures, while did not alter the expression of CD209 (Fig. 2C). We concluded that simultaneous 5-HT_2B_ agonism, in the presence of inflammatory activation signals, prevents the acquisition of a mature, activated phenotype in human CD1a^+^ dendritic cells.

### Specific activation of 5-HT_2B_ inhibits inflammatory responses in polyI:C-stimulated moDCs

**Figure 3:**
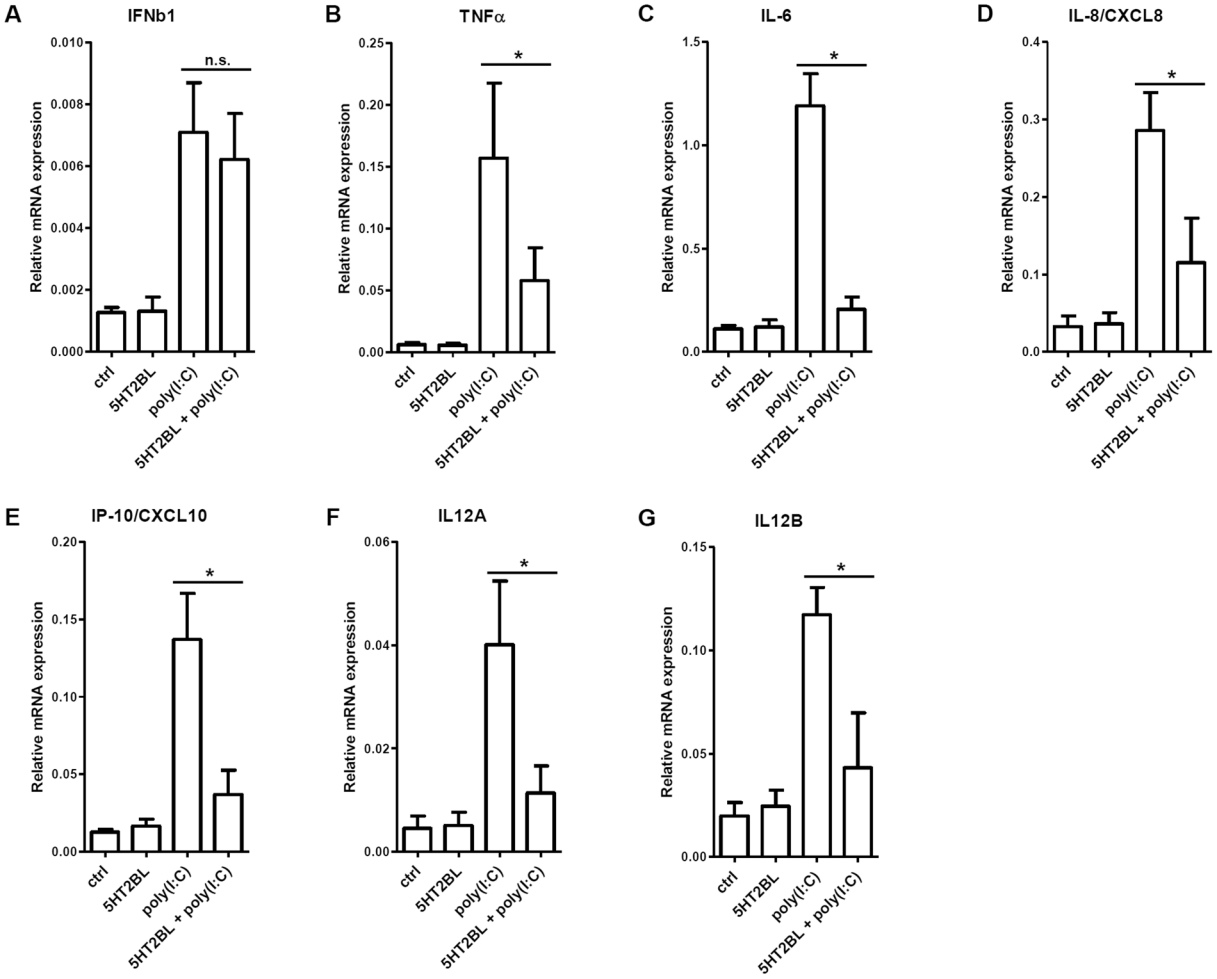
Specific 5-HT_2B_ stimulation interferes with the inflammatory response of human dendritic cells. Human CD1a^+^ moDCs were activated by 20 µg/ml polyI:C and/or 100 µg/ml BW723C86 (5HT2BL), and the expressions of inflammatory cytokine, chemokine, and type I interferon genes were assessed by Q-PCR. Relative mRNA expression data of triplicate measurements of six independent donors are shown as Mean ± SEM. ‘ctrl’ = non-treated control; *shows statistical significance at *p* < 0.05; *n*.*s*. means ‘non-significant’.

**Figure 4:**
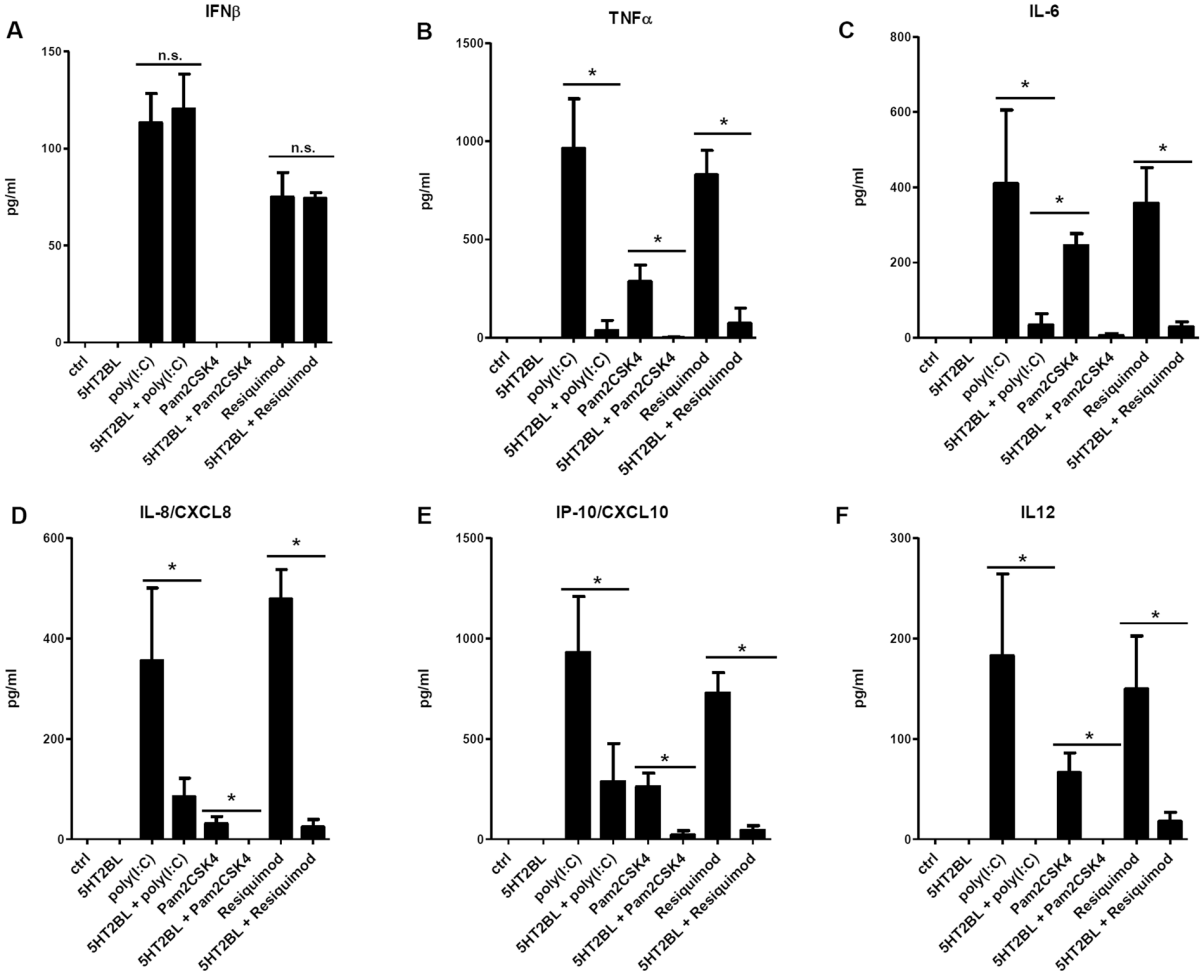
The ligation of 5HT_2B_ alters the cytokine profile of human moDCs upon inflammatory stimulation. Human CD1a^+^ moDCs were activated by 20 µg/ml polyI:C (TLR3 agonist), 10 ng/ml Pam2CSK4 (TLR2 ligand), 10 µg/ml Resiquimod (TLR7/8 agonist) and/or 100 µg/ml BW723C86 (5HT2BL). Inflammatory cytokine, chemokine, and type I interferon production was detected by ELISA in culture supernatants as detailed in Methods. Results represent the Mean ± SEM of triplicates of six (TLR3 activation) or three (TLR2 and TLR7/8 activation) independent donors. *p < 0.05; *n*.*s*. means ‘non-significant’.

**Figure 5:**
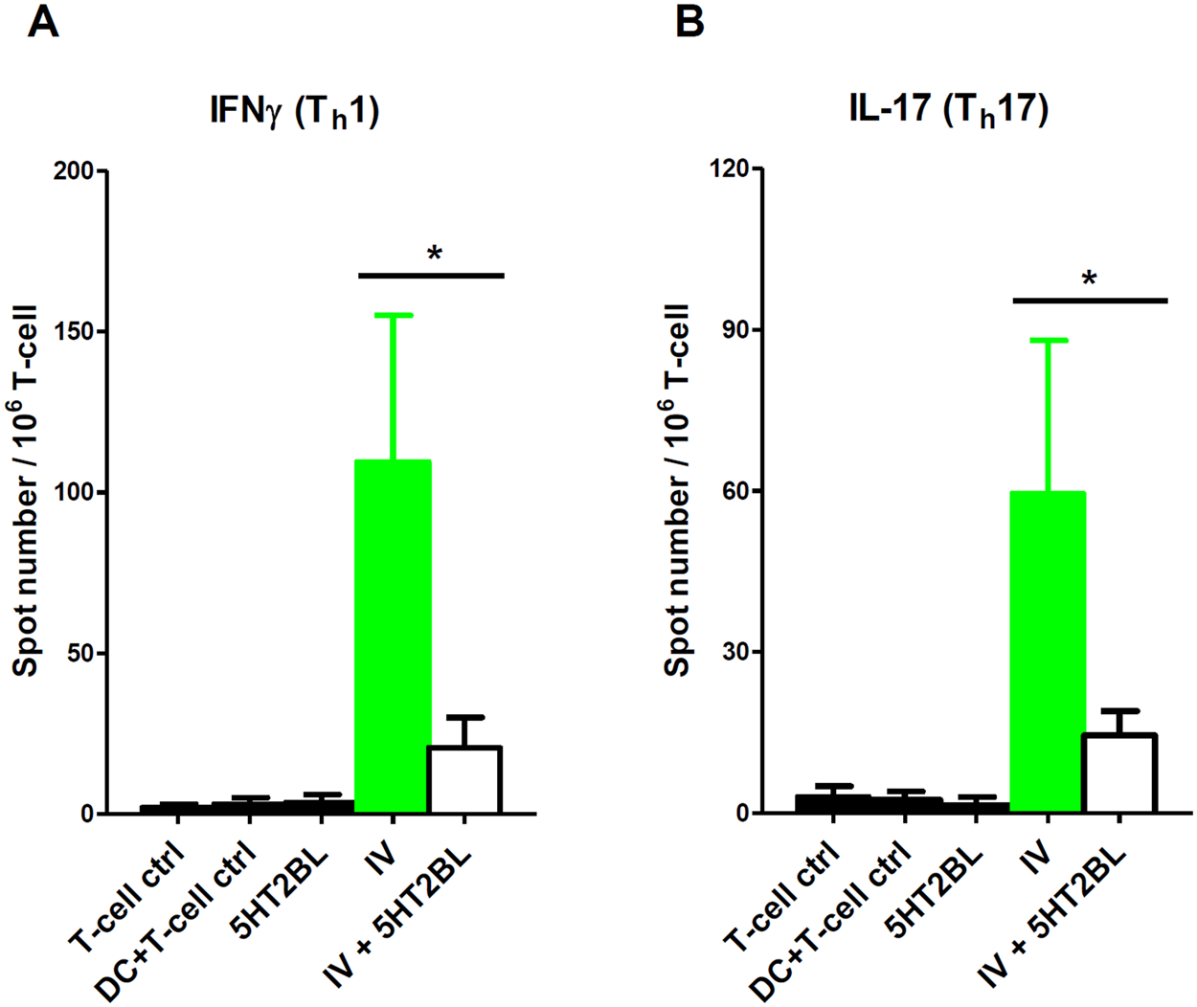
Activation of the 5-HT_2B_ receptor of human moDCs inhibits inflammatory adaptive immune responses. Human CD1a^+^ moDCs were activated by inactivated influenza virus (IV) for 24 h, washed, and then co-cultured with naive autologous CD4^+^ T lymphocytes for 4 days. The number of primed, IFNγ or IL-17 secreting T cells was assessed by ELISPOT assay. T cells alone (T-cell ctrl) or cultured with immature DCs (DC + T-cell ctrl) were used as controls. Induction of IFNγ (**A**) or IL-17 (**B**) production of autologous naive CD4^+^ T cells primed by CD1a^+^ moDC loaded with inactivated influenza virus (IV) is shown. 100 µg/ml BW723C86 (5HT2BL) was added to the moDCs alone for 24 h (black bars), or in combination with influenza virus (IV + 5HT2BL; empty bars). Green bars represent influenza virus-only activation (IV). Data represent Mean + SEM values of triplicate measurements of six independent donors. Asterisk indicates statistical significance (*p* < 0.05).

We showed that 5-HT_2B_ is selectively expressed and functional in CD1a^+^ but not in CD1a^−^ moDCs, moreover, its expression can be enhanced by innate PRR stimulation (Figs 1 and 2). Based on these findings we next tested the effects of specific 5-HT_2B_ ligation on the cytokine profile of mature moDCs. Treatment of polyI:C-activated CD1a^+^ moDCs with BW723C86 strongly reduced both the mRNA (Fig. 3) and the secreted levels (Fig. 4) of the pro-inflammatory cytokines TNF-α and IL-6, the chemokines IL-8 and IP-10, and the T cell stimulating factor IL-12. Decreased expression levels of these soluble regulators were consistently lower in polyI:C and BW723C86 co-treated cells as compared to the polyI:C-activated control (Figs 3B–G and 4B–F). Additionally, gene expression and production of the type I interferon IFNβ was not affected by 5-HT_2B_ and TLR3 co-activation (Figs 3A and 4A). We found similar anti-inflammatory effects when applied two other TLR ligands, Pam2CSK4 (a selective TLR2 agonist), and Resiquimod (a TLR7/8 ligand). Co-activation of TLR2 or TLR7/8 with 5-HT_2B_ resulted in significant reduction of TNF-α, IL-6, IL-8, IP-10, IL-12 secretion by CD1a^+^ moDCs (Fig. 5). Consistent with our findings BW723C86 treatment of CD1a^−^ moDCs lacking 5-HT_2B_ resulted in no significant alterations in cytokine levels upon TLR stimulation (Figure S3). These results reflect on the strong inhibitory capacity of 5-HT_2B_ on inflammatory and chemokine responses in CD1a^+^ moDCs when co-administered with innate immune signals. Taken the high migratory potential and inflammatory nature of CD1a^+^ moDCs our results suggest that important functional activities of these cells can be intervened by 5-HT_2B_ ligands as pharmacological modulators.

### Activation of the 5-HT_2B_ receptor inhibits the capacity of dendritic cells to prime autologous naive T helper 1 (Th1) and T helper 17 (Th17) cells

We demonstrated that co-treatment of CD1a^+^ moDCs by 5-HT_2B_ and TLR3 ligands resulted in the suppression of innate inflammatory responses as well as in the downregulation of the T cell co-stimulatory molecules CD80, CD83, CD86, and the inhibition of IL-12 secretion (Figs 2–4). These results raised the possibility that 5-HT_2B_ agonism might interfere with adaptive immunity. Thus, next we sought to investigate the functional consequences of these cellular responses upon pathogenic challenge by monitoring moDC-mediated T cell polarization. Previous reports showed that increased serotonin signaling tone suppresses graft-versus-host disease and negatively regulates adaptive immune responses^23,24^. It is also known that the polarization of adaptive “inflammatory” Th1 and Th17 responses are induced by and based on the production of IFNγ and IL-17, respectively. These inflammatory CD4^+^ helper T cell types are known to strongly promote inflammation and recruitment of other inflammatory cell types to the target tissues. The essential role of Th1 and Th17 cells in the development of inflammatory conditions in both the central nervous system and peripheral tissues in the context of infection and autoimmunity has been studied in various animal models and also in clinical studies^25–27^. To test the hypothesis that the anti-inflammatory and phenotype-modifying effects of 5-HT_2B_ activation in moDCs results in an unique T cell response, we co-cultured naive autologous CD4^+^ T cells with pathogen-activated moDCs and measured the number of activated IFNγ (Th1) and IL-17 (Th17) secreting effector T cells by ELISPOT assay (Fig. 5). In this experimental setup the common infectious agent influenza virus, known to cause both severe alveolar and CNS inflammations, was used as an immune response-provoking agent^28,29^. Activation of moDCs with flu virus resulted in strong Th1 (Fig. 5A) and Th17 (Fig. 5B) responses, but both effector T cell read-outs were markedly affected when BW723C86 co-treatments were applied. The Th1 and Th17-priming capacity of dendritic cells in the presence of BW723C86 was significantly lower than in the abscence of the selective 5-HT_2B_ ligand (Fig. 5A,B). These results demonstrate that the ligation of 5-HT_2B_ can potently inhibit the processing and/or presentation of viral peptide antigens by moDCs, and thus block inflammatory Th1 and Th17 responses.

**Figure 6:**
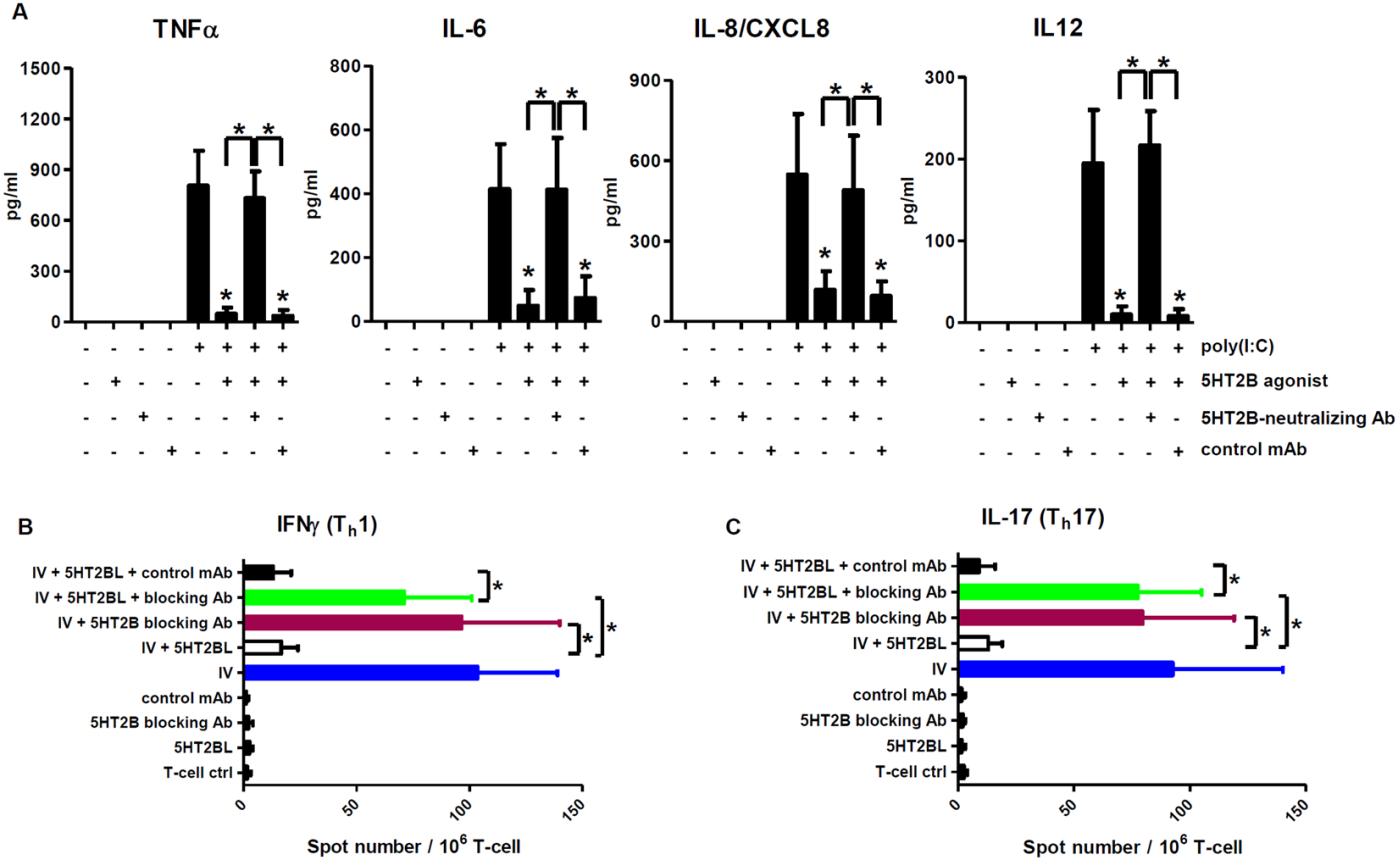
5-HT_2B_ receptor-neutralization abrogates the inhibitory effect of BW723C86 on the cytokine profile and Th1/Th17 cell-priming capacity of activated moDCs. Cells were co-cultured, activated, and the levels of secreted cytokines as well as the number of IFNγ or IL-17 secreting T cells was assessed by ELISA and ELISPOT as in Figs 4 and 5. (**A**) Non-treated, 100 µg/ml BW723C86, and 5-HT_2B_-neutralizing antibody-treated cells were used as negative controls. Cells treated with 20 µg/ml polyI:C served as positive controls. Co-treatments with polyI:C + BW723C86 were done as in Figs 3 and 4. 5-HT_2B_ receptor-neutralizing or isotype-matched monoclonal antibodies (control mAb) were always added to the cultures 30 minutes prior to activation. Supernatants of cultures were collected after 12 h and were measured by ELISA. Concentration of the secreted cytokines and chemokines are shown as Mean ± SEM values of three independent donors. (**B**–**C**) Blue bars represent influenza antigen-loaded DCs co-cultured with autologous naive CD4^+^ T cells (positive control). White bars show moDC co-activation with IV + 5HT2BL and subsequent T cell co-culturing. Claret bars (IV + 5HT2B blocking Ab) and green bars (IV + 5HT2BL + 5HT2B blocking antibody) demonstrate cultures that had been pre-treated with 5-HT_2B_ blocking antibody for 30 minutes prior to virus-loading (claret) or virus + BW723C86 co-treatment (green), respectively. DCs treated only with either 5HT2BL, the receptor-specific blocking antibody or an isotype-matched monoclonal antibody (control mAb), and co-cultured with CD4^+^ T cells were used as controls. Data represent Mean + SEM values of triplicate measurements of three independent donors. Asterisk shows statistical significance (*p* < 0.05).

After demonstrating the inhibitory effect of 5-HT_2B_ activation on the maturation (Fig. 2), inflammatory cytokine and chemokine production (Fig. 4), and CD4^+^ T cell-priming capacity of moDCs (Fig. 5), we aimed to check its contribution to the observed immunomodulatory phenomenon. We wanted to exclude any possible bystander effects from additional serotonin receptors thus we performed 5-HT_2B_ receptor-blocking experiments using an anti-5-HT_2B_ polyclonal antibody as written in Methods. Prior to this, we assessed the neutralizing capacity of the antibody and found that the optimal antibody concentration of blocking was 10 µg/ml; at this concentration the antibody was able to completely abolish Ca^2+^ signals evoked by 100 µg/ml BW723C86 (Figure S2). Thereafter, using the same cell activation protocols as in Figs 3 and 4, we found that specific neutralization of 5-HT_2B_ ablated the modulatory potential of BW723C86 on TNF-α, IL-6, IL-8, and IL-12 secretion upon polyI:C activation (Fig. 6A). We also tested the effects of 5-HT_2B_ receptor-blocking on moDC-mediated helper T cell responses and found that blocking of 5-HT_2B_ resulted in significantly less inhibition of virus-provoked Th1 (Fig. 6B) and Th17 responses (Fig. 6C) by BW723C86 than in controls. These results confirmed the indispensable role of 5-HT_2B_ in the anti-inflammatory regulation of dsRNA/virus-activated CD1a^+^ moDCs.

Finally, to reflect on the possible physiological importance of our findings we also tested the expression of 5-HT_2B_ in resting and activated human blood-derived CD1c^+^ DCs, a subset that is considered phenotypically and functionally similar to CD1a^+^ cells^1,3^. We found that CD1c^+^ DCs express high baseline level of 5-HT2B mRNA as compared to monocytes which have been reported to express the receptor gene (Figure S4)^10^. Poly(I:C) stimulation significantly increased the gene expression of 5-HT_2B_ in CD1c^+^ DCs suggesting its possible role in the regulation of inflammatory responses (Figure S4). Furthermore, we investigated the effect of the natural 5-HT receptor agonist serotonin on the 5-HT_2B_ signaling and cytokine secretion of CD1a^+^ moDCs. We applied a physiologically relevant concentration of 5-HT (10 µM) as was reported previously in similar human monocyte- and moDC systems^10,11^. Interestingly, specific blockade of 5-HT_2B_ signaling by the neutralizing antibody did not interfere with serotonin-dependent changes in IC Ca^2+^ levels (Figure S5A); however it did alter the TNF-α and IL-6 production of CD1a^+^ cells when co-applied with polyI:C in a similar manner as BW723C86 influenced the cytokine profile of moDCs (Figure S5B).

## Discussion

Identification of DC subsets with unique functions is recently emerging as a new challenge in DC biology. Monocyte-derived DCs, developing under inflammatory conditions, support protection against pathogens whereas DC subsets differentiating under steady state have a pivotal role in the control and resolution of inflammation and tissue destruction^30,31^. This functional diversity is determined by the destination site of DC precursors and the subtype-specific and compartmentalized expression of PRRs. We previously compared the expression and functional activity of RLR family members in two human moDC subsets distinguished by the expression and activity of PPARγ which controls the expression of type I CD1 molecule^15^. CD1a^−^ and CD1a^+^ moDCs were shown to differ in the expression of RLRs and RLR-coupled signaling elements, type I IFN and pro-inflammatory cytokine production^18^, as well as in their potential to provoke inflammatory T-lymphocyte responses^16^, and have also been shown to greatly influence immune pathology in influenza virus infection^32^. Based on these previous findings CD1a^+^ moDCs are considered to be an inflammatory DC subset with high migratory capacity as compared to their non-inflammatory, non-migratory CD1a^−^ counterparts.

Human CD1 proteins are closely associated with the presentation of various lipid, glycolipid, and lipopeptide antigens for T cells^33^. Lipid-rich environments, such as the brain or the intestines, induce and maintain the differentiation of the CD1a^−^ tolerogenic phenotype^16,34^. Interestingly, the production and signaling of serotonin have also been linked to lipid metabolism^35,36^. Based on our findings that the 5-HT_2B_ receptor subtype is exclusively expressed (Fig. 1) and functional only in the inflammatory CD1a^+^ moDC subset (Fig. 2), furthermore, that it negatively regulates both innate and adaptive inflammatory responses (Figs 3, 4, 5 and 6), we hypothesize that 5-HT_2B_ has an important role in maintaining self-tolerance through myeloid cells. This hypothesis may be partly confirmed by our further findings showing that human blood-derived CD1c^+^ DCs also exhibit high baseline mRNA levels of the receptor that can be further increased by innate immune stimulation (Figure S3). As our experiments were performed in serum-free medium, the serotonin background effect, deriving from the naturally occuring 5-HT in serum, can be excluded^37^. When serotonin was added to the culture medium of CD1a^+^ moDCs at physiological concentrations in a receptor-neutralization setting, it exerted similar anti-inflammatory effects as the 5-HT_2B_-specific ligand BW723C86 (Figure S5). This anti-inflammatory effect was comparable to, although not as prominent as, the effects of BW723C86 (Figure S5B). Interestingly, blocking 5-HT_2B_ did not alter the Ca^2+^ signaling of 5-HT-activated moDCs (Figure S5A). This phenomenon might be the result of the activation of other serotonin receptor subtypes, such as 5-HT_1_, 5-HT_4_, and 5-HT_7_, which have been reported to be expressed by moDCs^11^. The “serotonin tone” constantly present in the lipid-rich tissue microenvironments of the brain and gut might contribute to the maintenance of a self-tolerogenic immune homeostasis via the 5-HT_2B_ of resident monocyte-derived DCs. Since the averaging immunological effects of serotonin through 5-HT receptors may strongly vary depending on the given cell or tissue type, moreover intracellular 5-HT signals can compete and/or synergize with each-other, assessing the *in vivo* outcome of selective 5-HT_2B_ modulation in different tissue environments requires further investigation.

Although the expression of 5-HT_2B_ has been described in human monocyte-derived macrophages^7^ and in mouse CD11b^+^ mononuclear phagocytes^38^, this is the first report on characterizing the expression and function of 5-HT_2B_ in resting and activated human moDCs. To test the hypothesis that 5-HT_2B_ receptor activation may have impact on immune cell functions we studied the effects of the selective agonist BW723C86 on the cytokine profile of

activated moDCs. The applied working concentration of BW723C86 (100 µg/ml) is consistent with previous *in vivo* murine experimental setups^39,40^. In our experiments we used specific agonists of the innate immune receptors TLR2, TLR3, and TLR7/8. We showed that BW723C86 treatments potently inhibited proinflammatory cytokine and chemokine (TNF-α, IL-6, IL-8, IP-10) expression in human CD1a^+^ moDCs stimulated by these TLR ligands while also had an inhibitory effect on the production of the T cell modulatory cytokine IL-12 (Figs 3 and 4). Furthermore, 5-HT_2B_ agonism also interfered with the activation and polarization of naive T-lymphocytes toward Th1 and Th17 effector cells when co-cultured with influenza-virus loaded human moDCs (Fig. 5). We propose that this adaptive immune effect is the result of the 5-HT_2B_-mediated downregulation of T cell co-stimulatory molecules (Fig. 1) and the simultaneous inhibition of IL-12 secretion (Figs 3 and 4). Our results are in good agreement with reports showing that 5-HT_2B_ activation results in modulation of monocyte-derived macrophage differentiation to acquire the anti-inflammatory M2 phenotype^7^, as well as inhibition of lymphocyte proliferation and activation in various inflammatory and autoimmune pathologies (reviewed in^41^). Our results also demonstrate for the first time that specific activation of 5-HT_2B_ inhibits the polarization of human moDC-primed CD4^+^ T helper cells towards inflammatory Th1 and Th17 effector lymphocytes in infectious/inflammatory settings. Since Th1 and Th17 cells and their cytokines are key players in the etiology and symptomatology of various chronic inflammatory and autoimmune diseases of the CNS and other tissues^25,26^, these findings might be of particular importance. We speculate that the biochemical background of this phenomenon might be based on 5-HTR-PRR receptor cross-talk, competition for common adaptors and downstream signaling elements, such as TNF receptor-associated factor 3 (TRAF3) and TRAF6, and disruption of isochronous feedback loops of regulatory cytokines^42^.

The mobilization of innate and adaptive immune mechanisms is also well established in many psychiatric and neurological disorders^43–45^. It is an increasingly accepted hypothesis in neuropsychiatric research that a number of diseases affecting large populations, such as Alzheimer’s (AD), Parkinson’s disease, Major depression (MDD) are caused by chronic inflammation of the central nervous system. High resolution GWA studies found significant correlations between gene polymorphisms of innate immune receptors and the frequency of late onset AD^46,47^. It has also been reported in mice that the ligand specific activation of the mother’s TLRs and RLRs by lipopolysaccharide and polyI:C leads to cognitive deficits, decreased neurogenesis, and a marked increase in the appearance and deposition of Aβ aggregates in the brain of the offspring^48,49^. Since blood monocytes were shown to be able to translocate to the CNS^50^, our results may expand the role of CD1a^+^ moDCs to a more global context by suggesting the importance of 5-HT_2B_ signaling dysregulation and/or aberrant expression under autoimmune or infectious inflammatory conditions of the brain. Furthermore, platelet-derived serotonin has recently been shown to be critically involved in the pathology of allergic airway inflammation and aberrant levels of serotonin has been linked to defective T helper lymphocyte-priming by bone marrow DCs^51^. This may further broaden the possible patophysiological implications of our study to the biology of allergy and hypersensitivity reactions. Furthermore, the differentiation of brain-infiltrating monocytes into moDCs is considered to be essential in regulating CNS inflammation following trauma or infections^52,53^. Brain-resident moDCs have been reported to control reactive T-lymphocyte influx influencing neurodegenerative processes in various settings^54^. MoDCs are also thought to have a pivotal role in brain immunosurveillance contributing to the inflammatory aspects of neuropathologies associated with Alzheimer’s and Parkinson’s disease^55^. Our results suggest that the 5-HT_2B_ expressing CD1a^+^ DC subpopulation may play a crucial role here by downregulating inflammatory processes and controlling inflammatory helper T-cell activation. The immunomodulatory role of CD1a^+^ moDCs based on their 5-HT_2B_ receptor expression may also be important in the lungs and the gut where the environment is rich in serotonin, and thus this DC subtype represents an ideal target for drug design. An important limitation here, however is that the chronic administration of highly specific 5-HT_2B_ ligands are known to pose significant risk by causing cardiotoxic side-effects in mammals. Nonetheless, targeting 5-HT receptors in acute and chronic illnesses is an emerging topic in biomedicine, and identification of specific cell subsets and cellular functions that are associated with their signaling will be increasingly important to better understand the extremely complex patophysiology of serotonin^56^.

In conclusion, based on our findings we suggest that 5-HT_2B_ represents an important brake mechanism intrinsic to inflammatory antigen-presenting cell (APC) subsets, such as CD1a^+^ moDCs, that prevent excess inflammation, autoimmunity and consequent tissue destruction. Our results greatly expand the biological role of serotonin and 5-HT_2B_ which may act not only as neuromodulators, but also as important regulators of both innate and adaptive immune responses within the context of monocyte-derived APCs. Thus, 5-HT_2B_, as an immune modulator, emerges as a promising candidate in future pharmacological studies in both acute and chronic inflammatory and autoimmune diseases, and neuropsychiatric disorders.

## Methods

### Cell types, isolation, culturing and phenotyping

Leukocyte-enriched buffy coats were obtained from healthy blood donors drawn at the Regional Blood Center of the Hungarian National Blood Transfusion Service (Debrecen, Hungary) in accordance with the written approval of the Director of the National Blood Transfusion Service and the Regional and Institutional Ethics Committee of the University of Debrecen, Faculty of Medicine (Debrecen, Hungary). Written informed consent was obtained from the donors prior blood donation, and their data were processed and stored according to the directives of the European Union. Peripheral blood mononuclear cells (PBMCs) were separated by a standard density gradient centrifugation with Ficoll-Paque Plus (Amersham Biosciences, Uppsala, Sweden). Monocytes were purified from PBMCs by positive selection using immunomagnetic cell separation with anti-CD14 microbeads according to the manufacturer’s instruction (Miltenyi Biotech, Bergisch Gladbach, Germany). After separation on a VarioMACS magnet, 96–99% of the cells were CD14^+^ monocytes as measured by flow cytometry. Monocytes were cultured in 12-well tissue culture plates at a density of 2 × 10^6^ cells/ml in serum-free AIMV medium (Invitrogen, Carlsbad, CA) supplemented with 80 ng/ml GM-CSF (Gentaur Molecular Products, Brussels, Belgium) and 100 ng/ml IL-4 (Peprotech EC, London, U.K.) for five days. On day 2, the same amounts of GM-CSF and IL-4 were added to the cell cultures with changing 1/2 of their media. Cells were used for experiments on day 5 when they displayed DC phenotype as confirmed by flow cytometry analysis. Human CD1c (BDCA-1)+ dendritic cells were isolated using a Miltenyi Cell Isolation Kit in accordance with the manufacturer’s protocol (Miltenyi Biotech).

Phenotyping of resting and activated moDCs was performed by flow cytometry using anti-CD80-FITC, anti-CD83-FITC, anti-CD86-PE, anti-CD209-PE, anti-CD1a-PE, anti-CD14-PE (Beckman Coulter, Hialeah, FL), anti-HLA-DR-FITC and isotype-matched control antibody (Ab) (BD Pharmingen). Fluorescence intensities were measured by FACS Calibur (BD Biosciences, Franklin Lakes, NJ), data were analyzed by the FlowJo software (Tree Star, Ashland, OR). CD1a^+^ and CD1a^−^ moDC subsets were separated by FACS Aria III high-speed cell sorter (BD Biosciences) with higher than 98% purity in each case. Autologous naive T cells were separated from mononuclear cells of the same donor using a human naive CD4^+^ T Cell Isolation Kit (Miltenyi Biotech).

### Activation of monocyte-derived dendritic cells

High molecular weight polyinosinic:polycytidylic acid (polyI:C), Pam2CSK4 (TLR2 ligand), and Resiquimod (TLR7/8 agonist) were used at a working concentrations of 20 µg/ml, 10 ng/ml, and 10 µg/ml, respectively (all from InvivoGen, San Diego, CA). The high affinity, selective 5-HT_2B_ receptor agonist BW723C86 hydrochloride (Tocris Bioscience, Bristol, UK) was used at working concentrations of 1–300 µg/ml. The natural endogenous 5-HT receptor agonist serotonin (hydrochloride salt) was used at 1–100 µM concentrations (Tocris). Purified and inactivated A/Brisbane/59/7 H1N1 serotype influenza virus (kindly provided by the National Center for Epidemiology, Budapest, Hungary) of 1 × 10^7^ PFU/mL was used for *in vitro* treatment of 1 × 10^6^ per mL DC in serum-free AIMV medium for 24 hours. Virus-loaded cells were washed two times in sterile medium and then co-cultured with autologous naive T cells for ELISPOT assay.

To prepare cell lysates for western blotting cultures were sampled after 24 h of activation. To collect supernatants for ELISA or samples for flow cytometry cells were activated for 12 h or 24 h. Cell lysates were made after 8 h for QPCR measurements (if not stated otherwise).

### RNA isolation, cDNA synthesis and QPCR

RNA was isolated from moDCs using TRIzol reagent (Invitrogen) according to manufacturer’s protocol. The yield was measured by UV photometry on NanoDrop1000 instrument (ThermoFisher Scientific). RNA integrity was checked on Agilent BioAnalyzer (Agilent Technology) and samples with RIN > 7 were included in the further analysis. 1 µg RNA from each sample was used to generate first strand cDNA using High Capacity cDNA Reverse Transcription Kit (Applied Biosystems, Foster City, CA). Expressions of 96 genes were simultaneously measured on custom TaqMan Low Density Arrays by ABI HT7900 instrument (Applied Biosystems). Separate gene expression measurements were performed using specific TaqMan gene expression assays (Applied Biosystems). ΔCt method was used for normalization and PPIA (Cyclophilin A) was set as reference gene. Statistical analysis was performed in Graphpad Prism software and non-parametric Wilcoxon paired test was applied to compare the different conditions and identify significantly different expression patterns. Significant difference was considered at *p* < 0.05.

### Cytokine measurements

Culture supernatants were harvested 24 hours after activation and the concentrations of IFNβ, IL-6, TNFα, IL-8, IL-12, and IP-10 cytokines were measured using OptEIA kits (BD Biosciences, San Jose, CA) following the manufacturer’s recommendations. The precision of the kits were the following: Intra-Assay variation: CV < 10%; Inter-Assay variation: CV < 12% (CV% = SD/meanX100).

### Western blotting

Cells were lysed in Laemmli buffer and the protein extracts were tested by Ab specific for 5-HT_2B_ receptor (Abcam, Cambridge, UK), and β-actin (Sigma-Aldrich, St. Louis, MO, USA) diluted at 1:500 and 1:000, respectively. Anti-mouse Ab conjugated to horseradish peroxidase (GE Healthcare, Little Chalfont Buckinghamshire, UK) was used as the secondary Ab at a dilution of 1:5000. The SuperSignal enhanced chemiluminescence system was used for probing target proteins (Thermo Scientific, Rockford, IL). After the membranes had been probed for 5-HT_2B_, they were stripped and re-probed for β-actin.

### ELISPOT assay

Activated, virus-loaded DCs (2 × 10^5^ cells/well) were co-cultured with naïve autologous CD4^+^ T cells (10^6^ cells/well) in serum-free AIMV medium for 4 days at 37 °C in humidified atmosphere containing 5% CO_2_. Non-treated DC + T cell co-cultures and T cells without DC served as negative controls. Detection of activated, cytokine secreting CD4^+^ T cells producing IFNγ or IL-17 was performed by the avidin-horseradish peroxidase system (NatuTec, Frankfurt am Main, Germany). Plates were analyzed in an ImmunoScan plate reader (CTL Ltd., Shaker Heights, OH).

### Measurement of intracellular Ca^2+^ signaling

Fully differentiated human moDCs were seeded into 96-well plates at 5 × 10^5^ cell per mL in serum-free AIMV medium. Alterations in intracellular Ca^2+^ concentration by the 5-HT_2B_ agonist BW723C86 were assessed by the Fluo-8 No Wash Calcium Assay kit (Abcam) according to the manufacturer’s instructions. Fluorescence intensities were measured in a TriStar² LB 942 Multidetection Microplate Reader (Berthold Technologies, Bad Wildbad Germany). Samples were excited at 485 nm and Ca^2+^-bound Fluo-8 photon emission was recorded at 525 nm. Relative Fluo-8 fluorescence was expressed as F_max_/F_0_ (F_max_ = maximum fluorescence; F_0_ = baseline fluorescence).

### Cellular viability assays

The percentage of apoptotic cells was assessed by using an Annexin V apoptosis kit (BioVision, CA, USA) following the manufacturer’s recommendations. Necrotic cell death was also monitored based on the loss of membrane integrity and the uptake of propidium iodide (PI). Upon stimulation cells were harvested and stained with PI (10 μg/ml) and analyzed immediately by flow cytometry.

### 5HT_2B_ receptor neutralization

To block 5-HT_2B_ receptors, cells were treated with 10 µg/ml of anti-5-HT_2B_ polyclonal antibody (LifeSpan BioSciences, Seattle, WA) or an IgG isotype-matched control antibody (Biolegend, San Diego, CA) for 30 minutes prior to activation with the receptor agonist BW723C86 and/or poly(I:C). In case of ELISPOT experiments, cells were washed two times with sterile, serum-free AIMV medium before co-culturing with T cells.

### Statistical analysis

Data are presented as mean ± SEM. A *t-test* was used for comparison of two groups followed by Bonferroni correction. Two-way ANOVA was used for multiple comparisons. Differences were considered to be statistically significant at *p* values < 0.05 (*).

## Electronic supplementary material

Supplementary Information
