# Supplementary material for: Immunomodulatory capacity of the serotonin receptor 5-HT2B in a subset of human dendritic cells
Source: Sci Rep. 2018 Jan 29;8:1765. doi: 10.1038/s41598-018-20173-y (PMC5788853; doi:10.1038/s41598-018-20173-y)
Supplement: Supplementary file 1 — Supplementary Information [file 41598_2018_20173_MOESM1_ESM.pdf]

Immunomodulatory capacity of the serotonin receptor 5-HT2B in a subset of human dendritic cells

Attila Szabo[1,2,3,*], Peter Gogolak[3], Gabor Koncz[3], Zsofia Foldvari[3], Kitti Pazmandi[3], Noemi Miltner[3], Szilard Poliska[4], Attila Bacsi[3], Srdjan Djurovic[5,6], Eva Rajnavolgyi[3]

[1]NORMENT, KG Jebsen Centre for Psychosis Research, Institute of Clinical Medicine, University of Oslo, Oslo, Norway

[2]Division of Mental Health and Addiction, Oslo University Hospital, Oslo, Norway

[3]Department of Immunology, Faculty of Medicine, University of Debrecen, Debrecen, Hungary

[4]Department of Biochemistry and Molecular Biology, Faculty of Medicine, University of Debrecen, Debrecen, Hungary

[5]NORMENT, KG Jebsen Centre for Psychosis Research, Department of Clinical Science, University of Bergen, Bergen, Norway

[6]Department of Medical Genetics, Oslo University Hospital, Oslo, Norway

***Corresponding author:** Attila Szabo, PhD; NORMENT, KG Jebsen Centre for Psychosis Research, Oslo University Hospital, Building 49, Ullevål, Kirkeveien 166, PO Box 4956, Nydalen, 0424 Oslo, Norway; E-mail: attila.szabo@medisin.uio.no

Supplementary Figure 1

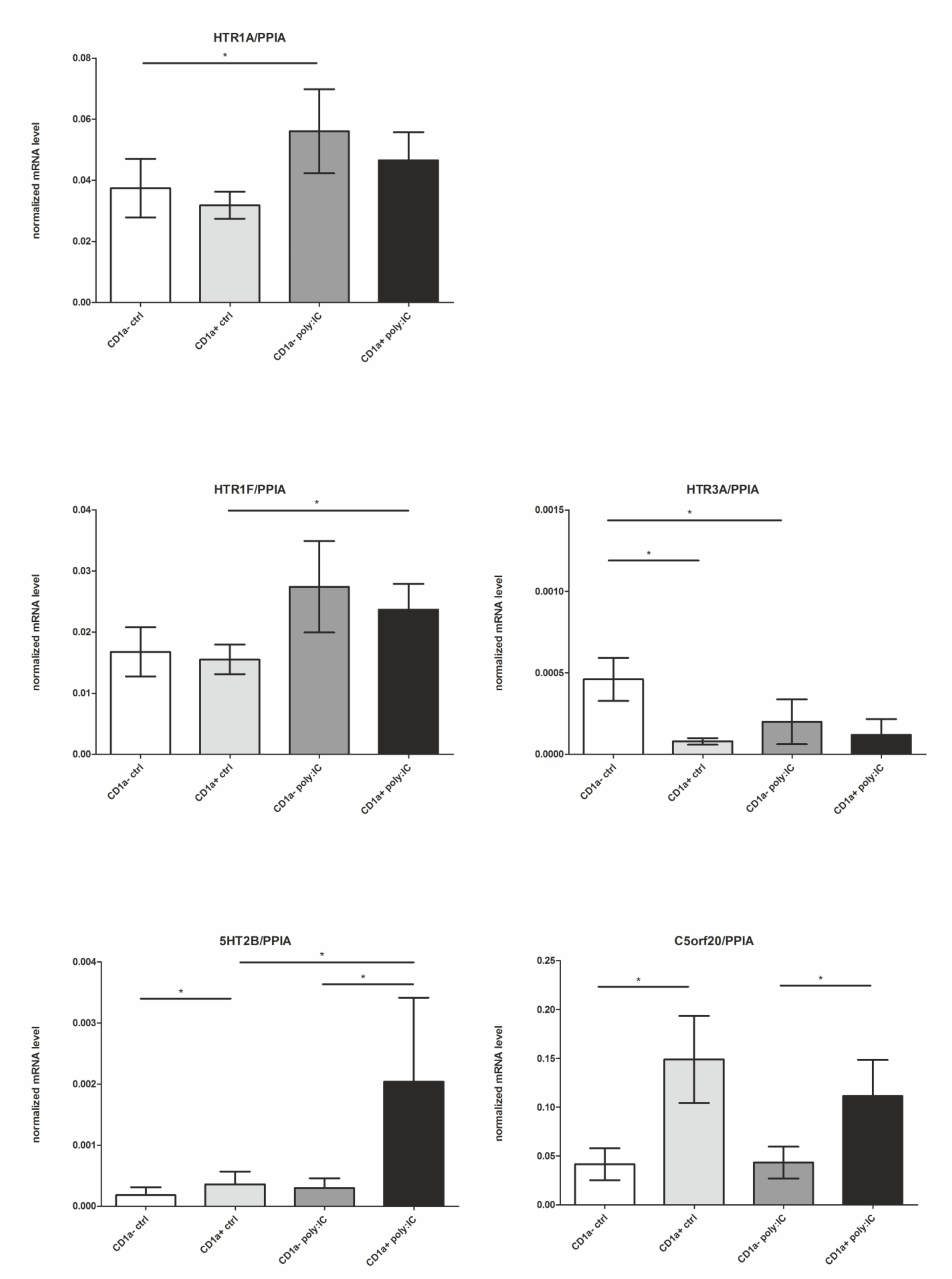

Supplementary Figure 2

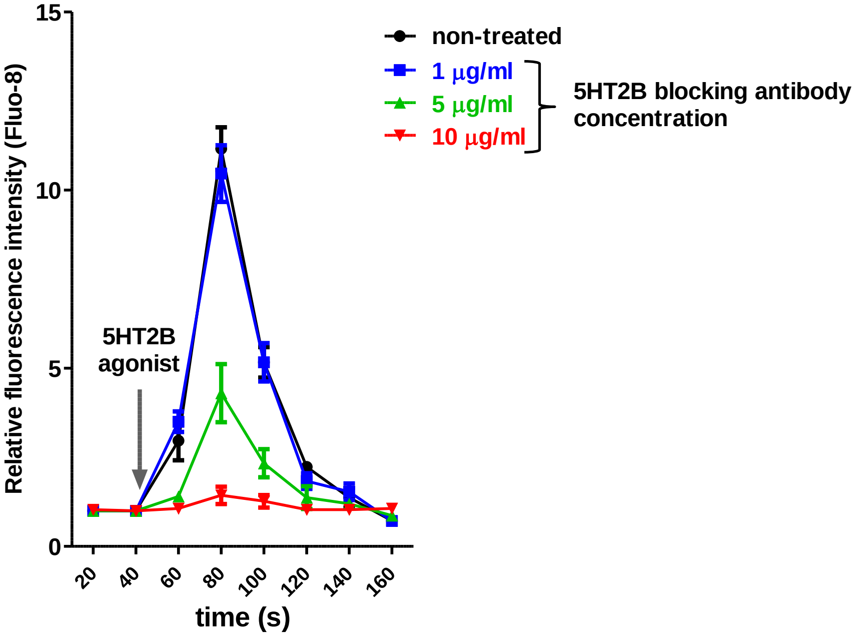

Supplementary Figure 3

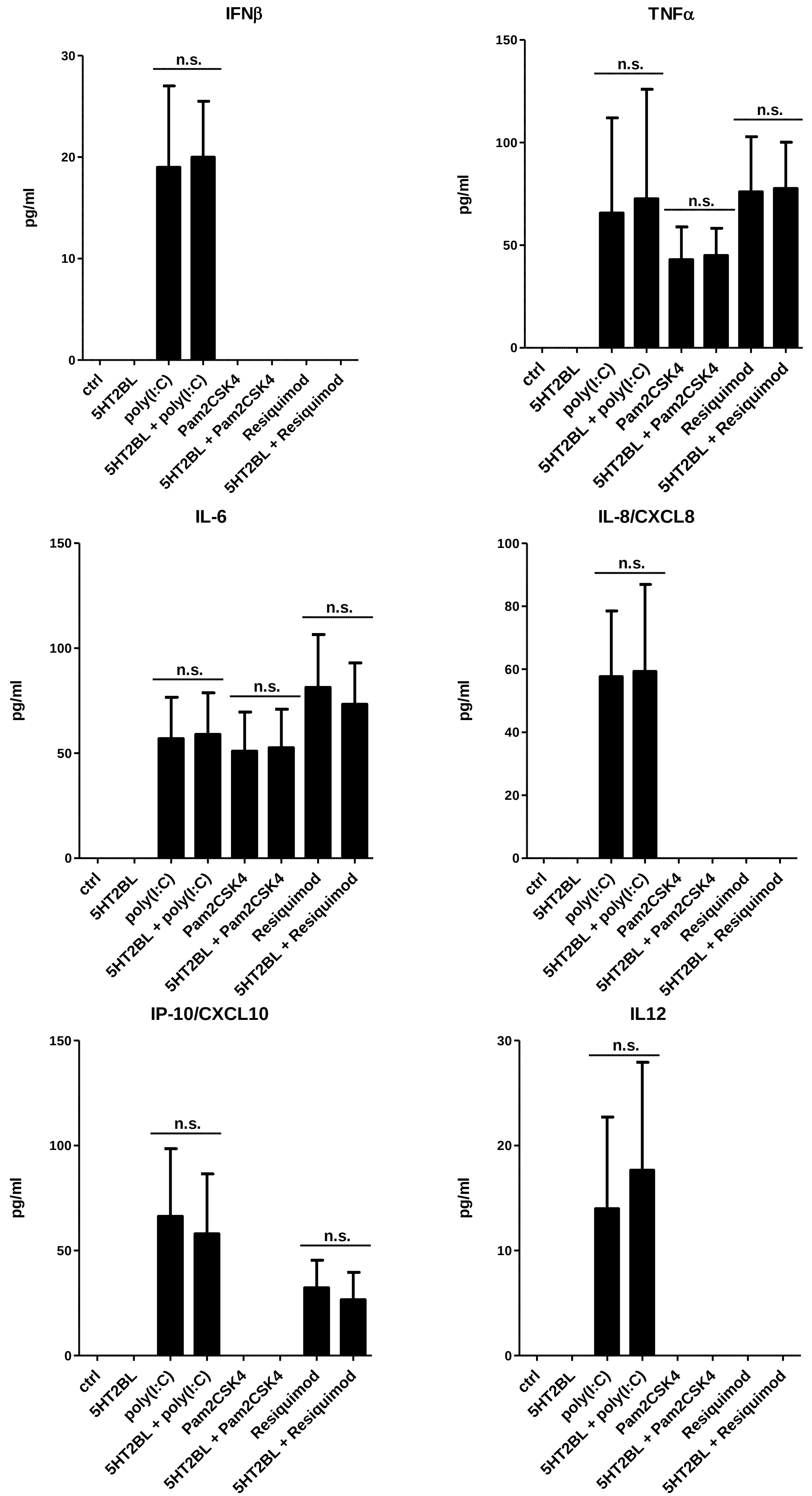

Supplementary Figure 4

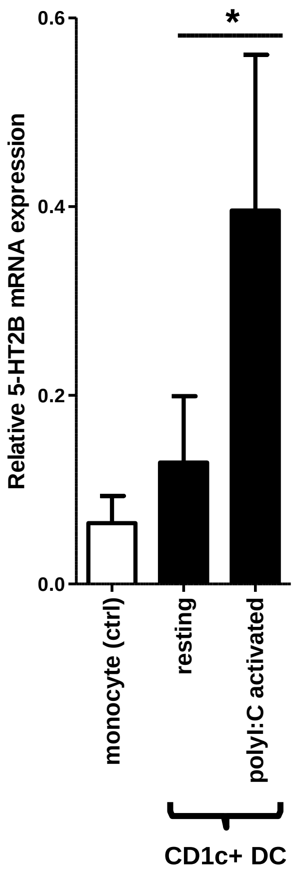

Supplementary Figure 5

A

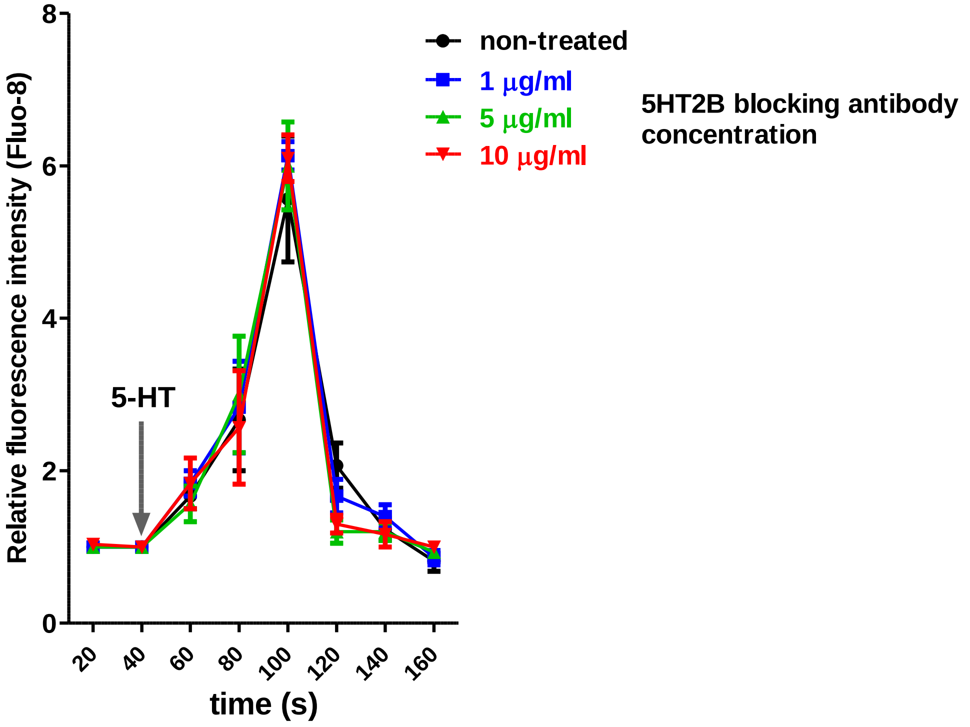

B

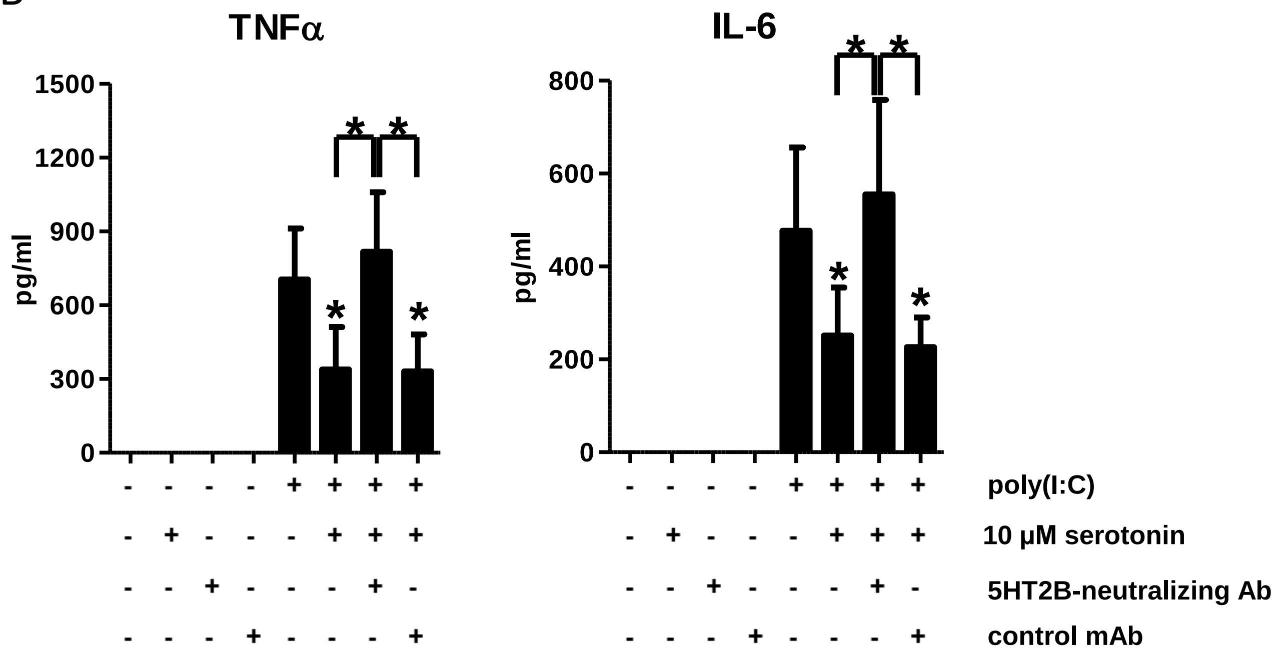

SUPPLEMENTARY FIGURES

Figure S1

Gene expression of serotonin receptors and DC-associated factors in human dendritic cell subsets at baseline and following polyI:C activation

Realtive mRNA expression data were extracted from the heat map in Figure 1A. All of the genes that displayed statistical difference in its expression profile in $CD1a^-$ versus $CD1a^+$ moDCs are shown here. Mean ± SEM values of five independent donors are presented. Asterisk means $p < 0.05$.

Figure S2

Kinetics of $5\text{-}HT_{2B}$ receptor-neutralization

Relative Fluo-8 fluorescence intensity was measured as written in Methods. $CD1a^+$ moDCs were pre-treated with 1-10 µg/ml $5\text{-}HT_{2B}$ receptor-blocking antibody 30 minutes prior to activation by 100 µg/ml BW723C86. Data of triplicate measurements of three independent donors are represented as Mean ± SEM.

Figure S3

$5HT_{2B}$ agonist treatment did not influence the TLR-evoked inflammatory response in human $CD1a^-$ moDCs

Human $CD1a^-$ moDCs were activated by 20 µg/ml polyI:C (TLR3 agonist), 10 ng/ml Pam2CSK4 (TLR2 ligand), 10 µg/ml Resiquimod (TLR7/8 agonist) and/or 100 µg/ml BW723C86 (5HT2BL). Inflammatory cytokine, chemokine, and type I interferon production was detected by ELISA in culture supernatants as detailed in Methods. Results represent the Mean ± SEM of triplicates of three independent donors. *n.s.* means 'non-significant'.

Figure S4

Gene expression of $5\text{-}HT_{2B}$ in resting and activated human blood-derived $CD1c/BDCA\text{-}1^+$ dendritic cells

Monocytes and $CD1c^+$ dendritic cells were isolated from human blood as written in Methods. Monocytes from the same donor were used as positive control (ctrl). $CD1c^+$ DCs were activated by 20 µg/ml polyI:C for 8h. Relative $5\text{-}HT_{2B}$ mRNA levels were assessed by QPCR. Expression data of triplicate measurements of three independent donors are shown as Mean ± SEM. * shows statistical significance at $p<0.05$

Figure S5

Serotonin effects on 5-HT_{2B} neutralization in $CD1a^+$ moDCS

(A) Relative Fluo-8 fluorescence intensity was measured as written in Methods. $CD1a^+$ moDCs were pre-treated with 1-10 μg/ml 5-HT_{2B} receptor-blocking antibody 30 minutes prior to activation by 10 μM serotonin hydrochloride. Data of triplicate measurements of three independent donors are represented as Mean ± SEM. **(B)** Cells were activated and cytokine levels were assessed as in Figure 6. Non-treated, 10 μM serotonin hydrochloride-treated, and 5-HT2B-neutralizing antibody-treated cells were used as negative controls. Cells treated with 20 μg/ml polyI:C served as positive controls. Co-treatments with polyI:C+serotonin were done similarly as polyI:C+BW723C86 co-stimulations. 5-HT2B receptor-neutralizing or isotype-matched monoclonal antibodies (control mAb) were added to the cultures 30 minutes prior to activation. Supernatants of cultures were collected after 12h and were measured by ELISA. Concentration of the secreted cytokines and chemokines are shown as Mean ± SEM values of three independent donors.
